# Morphological Alterations in Gastrocnemius and Soleus Muscles in Male and Female Mice in a Fibromyalgia Model

**DOI:** 10.1371/journal.pone.0151116

**Published:** 2016-03-17

**Authors:** Gabriel Alejandro Bonaterra, Hanna Then, Lisa Oezel, Hans Schwarzbach, Matthias Ocker, Kati Thieme, Pietro Di Fazio, Ralf Kinscherf

**Affiliations:** 1 Anatomy und Cell Biology, Department of Medical Cell Biology, University of Marburg, Marburg, Hessen, Germany; 2 Institute for Surgical Research, Philipps University of Marburg, Marburg, Hessen, Germany; 3 Institute for Medical Psychology, Philipps University of Marburg, Marburg, Hessen, Germany; Institut de Myologie, FRANCE

## Abstract

**Background:**

Fibromyalgia (FM) is a chronic musculoskeletal pain disorder, characterized by chronic widespread pain and bodily tenderness and is often accompanied by affective disturbances, however often with unknown etiology. According to recent reports, physical and psychological stress trigger FM. To develop new treatments for FM, experimental animal models for FM are needed to be development and characterized. Using a mouse model for FM including intermittent cold stress (ICS), we hypothesized that ICS leads to morphological alterations in skeletal muscles in mice.

**Methods:**

Male and female ICS mice were kept under alternating temperature (4°C/room temperature [22°C]); mice constantly kept at room temperature served as control. After scarification, gastrocnemius and soleus muscles were removed and snap-frozen in liquid nitrogen–cooled isopentane or fixed for electron microscopy.

**Results:**

In gastrocnemius/soleus muscles of male ICS mice, we found a 21.6% and 33.2% decrease of fiber cross sectional area (FCSA), which in soleus muscle concerns the loss of type IIa and IIx FCSA. This phenomenon was not seen in muscles of female ICS mice. However, this loss in male ICS mice was associated with an increase in gastrocnemius of the density of MIF^+^ (8.6%)-, MuRF^+^ (14.7%)-, Fbxo32^+^ (17.8%)-cells, a 12.1% loss of capillary contacts/muscle fiber as well as a 30.7% increase of damaged mitochondria in comparison with male control mice. Moreover, significant positive correlations exist among densities (n/mm^2^) of MIF^+^, MuRF^+^, Fbxo32^+^-cells in gastrocnemius/ soleus muscles of male ICS mice; these cell densities inversely correlate with FCSA especially in gastrocnemius muscle of male ICS mice.

**Conclusion:**

The ICS-induced decrease of FCSA mainly concerns gastrocnemius muscle of male mice due to an increase of inflammatory and atrogenic cells. In soleus muscle of male ICS and soleus/gastrocnemius muscles of female ICS mice morphological alterations seem to occur not at all or delayed. The sex-specificity of findings, which is not easily reconciled with the epidemiology of FM (female predominance), implicate that gastrocnemius muscle of male ICS mice should preferentially be used for future investigations with FM. Moreover, we suggest to investigate morphological and/or molecular alterations at different time-points (up to two weeks) after ICS.

## Introduction

Fibromyalgia (FM) is a multifactorial disease being characterized by chronic pain, often accompanied by various psychological symptoms; however, pathophysiological mechanisms have not yet been identified until now. FM is characterized by symptoms like musculoskeletal pain, depression, fatigue, headache and sleep disturbances and is diagnosed according to the classification criteria established by the American College of Rheumatology (ACR) [1]. The diagnosis is not easy and may be frequently overlooked; this is why new diagnostic markers in FM are needed and its pathogenic mechanism remains elusive [2]. Pilot studies suggest that cytokines may play an important role in FM, because associations have been observed between cytokines and several symptoms common in FM [2,3]. However, mitochondrial dysfunction has recently been proposed as a relevant event in the pathogenesis of this disorder [4]. In this context, cells undergoing aerobic metabolism, produce reactive oxygen species (ROS) as a by-product of the mitochondrial electron transport chain; additionally, it has been demonstrated, for example that the mitochondrial coenzyme Q10 (CoQ10) is essential for mitochondrial chain efficiency, and evidence exists that it affects the expression of genes involved in inflammatory response [5]. Furthermore, FM may be associated with immune dysregulation by blood levels of pro-inflammatory cytokines, affecting the normal neuronal function of pain-related neurotransmitters [3]. A link between the inflammatory cytokine tumor necrosis factor-α (TNF-α), mitochondrial integrity and mitochondrial ROS levels would suggest that inflammation occurring in several FM patients may be due to mitochondrial dysfunction [2]. Mitochondrial dysfunction was found to be accompanied by caspase-1 activation and increased serum levels of pro-inflammatory cytokines like interleukin-1beta (IL-1β) and IL-18 [6]. Moreover, on the one hand skeletal muscle exhibits very high plasticity and is the major body protein reservoir. On the other hand muscle proteins can be metabolized into free amino acids under disease conditions (e.g. bed rest, immobilization), in starvation, and in many pathologic states (e.g. diabetes, cancer cachexia, sepsis, AIDS, burn injury, renal failure, trauma) [7–9], because muscle atrophy prevails in numerous diseases [10]. Muscle atrophy may either result from enhanced proteolysis or depressed protein synthesis, or both [8,11]. However, recent clinical and mechanistic studies have shown that increased proteolysis is the major determinant of muscle wasting in numerous catabolic states and of alterations in myopathies or dystrophies [11]. In this context, the muscle-specific E3 ligases muscle ring finger 1 (MuRF1) and muscle atrophy F-box (MAFbx)/Fbxo32/atrogin1 were found to be up-regulated in nearly all situations of muscle wasting; additionally, MuRF1 was shown to be overexpressed at the protein level under catabolic conditions [10,12,13]. Conversely, cytokines (i.e. TNF-α, IL-6), hormones (i.e. glucocorticoids), and myostatin up-regulated the ubiquitin–proteasome system (UPS) [11]. Although several trials have been approved to test current available drugs for FM treatment, attempts to develop a specific compound for FM have to be performed yet [14]. Thus, it is tempting to speculate that anti-inflammatory and/or anti-oxidative treatment might be useful for FM patients treatment. However, prior to that an adequate FM-animal model is needed for the identification of markers related to appropriate treatment and pharmacological therapy of FM. Several models have been proposed e.g., sound stress, mechanical hyperalgesia and SART (specific alternation rhythm of temperature) stress [15–17]. In this context Nishiyori and Ueda [14] improved an animal model for dysautonomia, referred as the specific SART model [15,18]. Nishiyori and Ueda [14] established this novel mouse model of FM using intermittent cold stress (ICS), which produces long-lasting thermal hyperalgesia and mechanical allodynia. Validation and additional support for the ICS animal model for FM have been shown in the last five years by several studies [19–21], however also most recently [22], using auxiliary tests for measuring allodynia, anxiety, behaviour, hyperalgesia.

The aim of our study was to investigate gender, morphological and/or molecular changes in skeletal muscle to potentially deliver the basis for new strategies to pharmacologically/therapeutically treat FM by using the extensively validated ICS-induced FM mouse model.

## Materials and Methods

### Animal treatments

The mouse model used in this study has been described by Nishiyori and Ueda [14,19,20]. Briefly, 30 six-weeks-old C57BL/6J mice (Charles River, Paris, France), male (n = 14) and female (n = 16) were used. These mice were kept at room temperature, and *ad libitum* feeding of a standard laboratory diet and tap water. The mice (male [n = 7], female [n = 8]) were kept at 4°C on the first day (day 0). The next morning (day 1), mice were transferred to the normal room (22°C) temperature for 30 min, afterwards mice were put in the cold room again for 30 min and these processes were repeated for 6 h. Mice were then left 4°C overnight. After the same treatments on the next morning (day 2), mice were finally taken out from the cold room on day 3 and were kept there for adaptation at least 1 h before the end of the experiment. Remaining mice (n = 15) served as control animals and were constantly kept at room temperature. Mice were placed on a stainless steel mesh and covered with Plexiglas cage or placed in 330 cm^2^ and 12 cm height Plexiglas cages with appropriate woody nesting material. At the end the mice were killed by cervical dislocation. Tissue of the right triceps surae (gastrocnemius and soleus) muscle was removed and snap-frozen in liquid nitrogen-cooled isopentane or immediately fixed in 100% ITO-solution for electron microscopy. The care and use of all mice in this study was carried out in accordance with institutional ethical guidelines for animal experiments and the study protocol complied with the institute’s guidelines and was approved by the Government of Hessen (V 54–19 c 20 15 (1) MR 20/19 Nr. 8/2012).

### Transmission electron microscopy (TEM)

Gastrocnemius and soleus muscles were cut into small pieces and fixed in 2.5% glutaraldehyde, 1.25% (para)formaldehyde (PFA), 2 mM picric acid (in 0.1 M cacodylate buffer ([pH 7.0]), afterwards post-fixed in 1% Osmium tetroxide, dehydrated with ethanol/propylene oxide, and embedded in glycid-ether 100 (EPON 812, SERVA Electrophoresis GmbH, Heidelberg, Germany) at a longitudinal orientation. The tissue was then cut using an Reichert Ultracut S ultramicrotome (Leica Microsystems, Wetzlar, Germany), and ultrathin sections (60–80 nm) were mounted on copper grids, contrasted with 4% uranyl acetate and lead citrate and observed with a Zeiss EM 10 C TEM (Carl Zeiss GmbH, Jena, Germany). Pictures were taken with Image a SP System (SYSPROG, Minsk, Belarus), from each section at a magnification of x 20,000 and analysed by computer software ImageJ (Scion Image, National Institutes of Health, Bethesda, USA). The degree of ultrastructural changes in the mitochondria was evaluated using three criteria: thickening of cristae, unfolding of cristae and double membrane integrity. The mitochondria were picked out in two levels of quality: normal or damaged and the results expressed as a percentage of the total mitochondria counted in 10–14 random pictures examined from each individual muscle.

### Immunohistochemistry

Cryostat sections (6–7 μm) of gastrocnemius or soleus muscle were fixed with 4% paraformaldehyde/phosphate buffered solution (PFA/PBS; 10 min, RT). Endogenous peroxidase was blocked with 3% H_2_O_2_. Non-specific sites were blocked with 1% normal porcine serum (Dako Deutschland GmbH, Hamburg, Germany) in PBS. Table A (in S1 File), shows the primary monoclonal and polyclonal antibodies used in this study.

Single staining was performed by incubation of the sections with a primary antibody and thereafter with goat anti-rabbit horseradish peroxidase (HRP)-conjugated (LINARIS GmbH, Dossenheim, Germany) or polyclonal goat anti-rat HRP-conjugated (AbD Serotec, Kidlington, UK) antibodies; endogenous peroxidase activity was suppressed with 3% H_2_O_2_ in PBS; afterwards the sections were incubated with with 3’-diaminobenzidine (DAB) solution (Roche Diagnostics). Nuclei were counterstained with Mayer's Hematoxylin (Carl Roth GmbH & Co. KG, Karlsruhe, Germany) or Hoechst 33342 (Invitrogen, Eugene, USA). Finally, sections were photographed using an Axioplan2 imaging microscope (Carl Zeiss GmbH) and the digital high resolution imaging system AxioCam/AxioVision (Carl Zeiss GmbH). The quantification of the immunohistochemistry (IHC) was performed using the AxioVision Release 4.8.2 software (Carl Zeiss GmbH) or the software ImageJ (Scion ImageJ, National Institutes of Health, Bethesda, USA).

### Lectin histochemistry

Cryo cross-sections of gastrocnemius or soleus muscle were cut in a cryostat, fixed in 4% PFA/PBS 10 min, endogenous peroxidase was blocked with 3% H_2_O_2_, and washed in phosphate-buffered saline (PBS; pH 7.4) at room temperature. Sections were then incubated 30 min at 37°C with 40 μg/ml in PBS HRP-conjugated Isolectin B_4_ of *Bandeiraea simplicifolia* (BSI- B_4_) (Sigma-Aldrich Co. LLC, ST. Louis, USA). Afterwards sections were washed with PBS and incubated with DAB solution (Roche Diagnostics), rinsed with PBS, counterstained with Mayer's hematoxylin (Carl Roth GmbH, Karlsruhe) and cover-slipped in mounting medium for histology, DePeX (SERVA Electrophoresis GmbH, Heidelberg, Germany). Nuclei were counterstained with Mayer's Hematoxylin (Carl Roth GmbH). Capillary with a diameter of ≤ 5μm were identified in cross-sections by brown staining and quantified using the software software ImageJ (Scion Image, National Institutes of Health, Bethesda, USA). The average capillary numerical density (number of capillaries/mm^2^) and capillary contact per muscle fiber (number of capillaries/fiber) was calculated for each muscle cross-section. To analyze neuromuscular junctions α-Bungarotoxin (α-BT) histochemistry was performed (Materials and methods A in S1 File).

### Adenosine triphosphatase (ATPase) staining

Serial transverse sections (6–7 μm) were cut in a cryostat at -20°C and stained for myofibrillar ATPase as previously described [23]. Three fiber types (I, IIa and IIx) could be distinguished. Microscopic images of the ATPase-stained cross-sections were recorded using an Axio Image.M2 microscope (Carl Zeiss GmbH) including the digital high-resolution imaging system AxioCam HRc/AxioVision Rel. 4.8 (Carl Zeiss GmbH). Digitalized figures were processed, arranged, and lettered by standard imaging software ImageJ. The fiber types (I, IIa and IIx) were individually counted and expressed as number of fibers/mm^2^. The cross-sectional area of each fiber type was determined and expressed in total area (μm^2^) of each fiber type.

### Real time quantitative reverse transcription polymerase chain reaction (qRT-PCR)

RNA was extracted from 1.0–2.0 mg tissue using peqGOLD Isolation Systems TriFast^™^ (PEQLAB Biotechnologie GmbH, Erlangen, Germany) according to the manufacturer’s specified protocols. RNA concentration and purity were determined by OD_260 nm_ and OD_280nm_ (OD_260 nm_ / OD_280nm_ = 1.7 to 2.0) using a NanoDrop 2000c spectrophotometer (Thermo Scientific, Schwerte, Germany). RNA integrity was confirmed by the lab-on-a-chip technology, using an RNA 6000 NanoChip kit on an Agilent 2100 Bioanalyzer (Agilent Technologies, Waldbronn, Germany). An aliquot of 0.7 μg total RNA was treated with 1 unit DNAse (Thermo Scientific, St. Leon-Rot, Germany; 30 min, 37°C). Reverse transcription of RNA was performed with 500 ng oligo (dT)_12–18_ primer, 20 units of the Affinity Script multiple temperature cDNA synthesis (Agilent) and 24 units of Ribo Lock^™^ RNAse inhibitor (Fermentas; 1h, 42°C) and 4 mM dNTP- Mix. The cDNA was used for qRT-PCR using the QuantiTect / primerAssays from QIAGEN GmbH (Hilden, Germany; Table B in S1 File).

Amplification and data analysis were performed using the Mx3005P^™^ QPCR System (Stratagene) including the relative standard curve method. For each unknown sample, the relative amount was calculated by linear regression analysis from their respective standard curves. For relative quantification, a standard curve was generated from a pool of cDNA. Specificity of the amplified product was confirmed by melting curve analysis (55°C–95°C), and additionally, by using a 2% agarose gel electrophoresis to approve the amplicon size in conjunction with melting curve data. The expression of Actb, Gapdh and Tbp (Table B in S1 File) were analyzed using the NormFinder software to ascertain the most suitable reference gene to normalize the RNA input as described earlier [24].

### Statistical analysis

Results are presented as means + standard error of the mean (SEM). Depending on the mode of distribution statistical procedures were performed by the Mann-Whitney U test or by the unpaired Student’s t-test, using SigmaPlot^®^ software (Systat Software, Inc, Chicago, USA). p< 0.05 was chosen for statistical significance. When appropriate, statistical analyses using ANOVA test were performed.

## Results

### Differences in fiber density and fiber cross sectional area (FCSA) in gastrocnemius and/or soleus muscles of male control- vs. ICS mice

**Fig 1:**
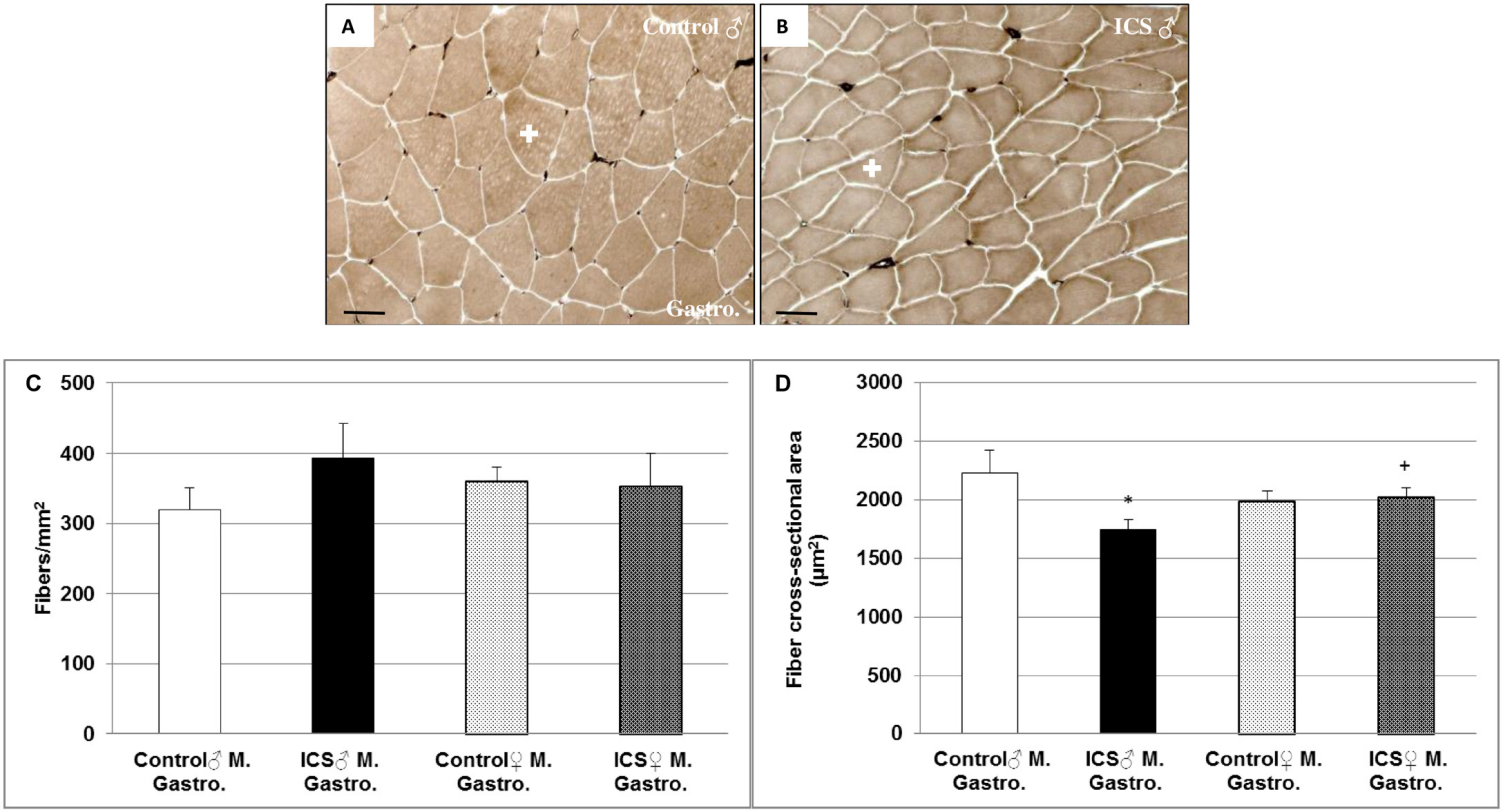
Fiber density (n/mm^2^) and fiber cross sectional area (μm^2^) of gastrocnemius (Gastro.) muscle. Representative images of gastrocnemius muscle cross sections stained for myofibrillar ATPase after pre-incubation at pH 4.6 of male (♂) control (**A**) and ICS (**B**) are shown. Quantification of fiber type density (**C**) and fiber cross sectional area (**D**) are shown. Type IIa (white cross); Values are given as mean + SEM; *P ≤ 0.05, significance vs control and ^+^*P* ≤ 0.05 vs male mice. N = 8–9 animals per group. Bar = 50 μm.

**Fig 2:**
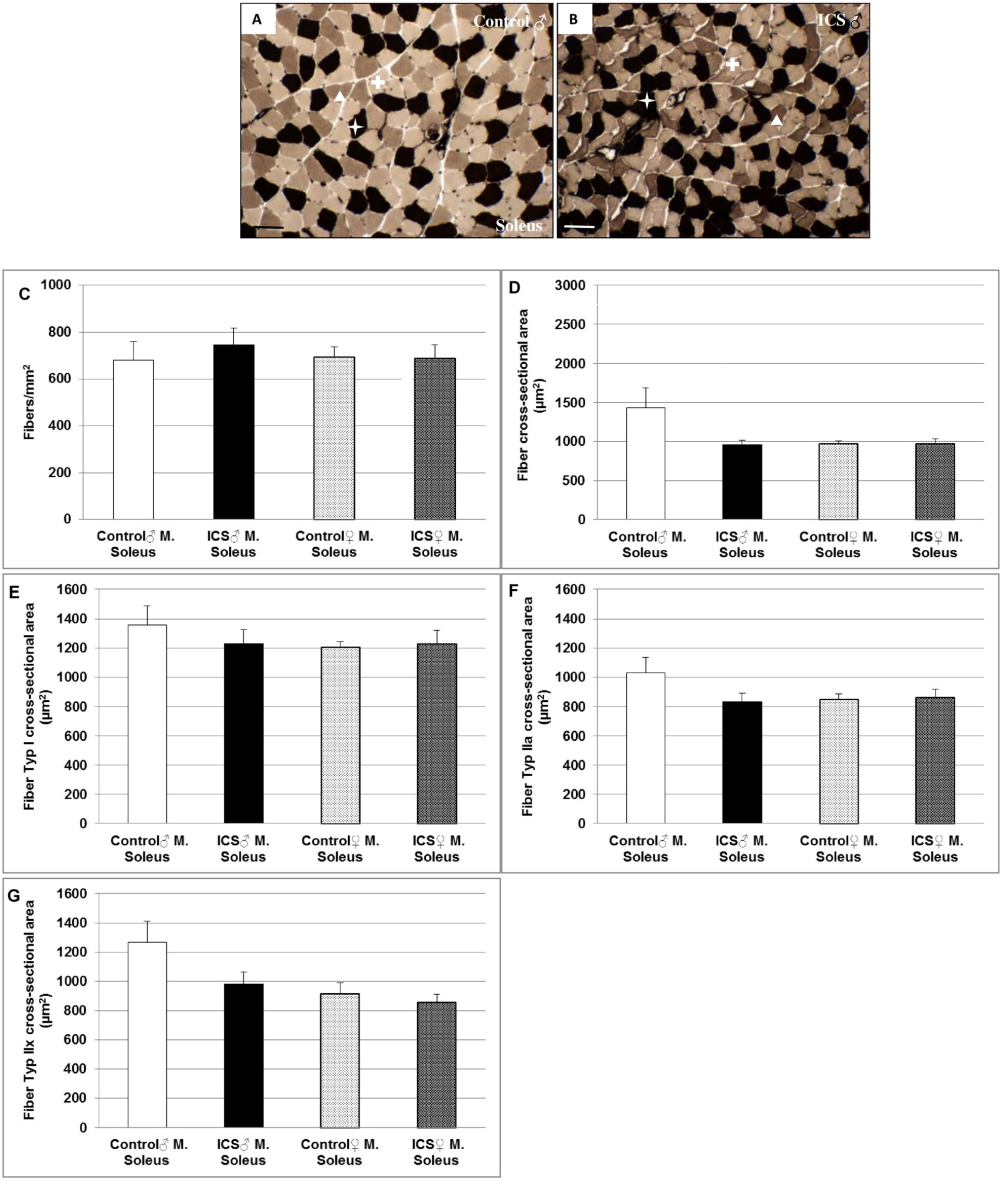
Fiber density (n/mm^2^) and fiber cross sectional area (μm^2^) of soleus muscle. Representative images of soleus muscle cross sections stained for myofibrillar ATPase after preincubation at pH 4.6 of male (♂) control (**A**) and ICS (**B**) mice are shown. Quantification of fiber density (**C**), fiber cross sectional area (FCSA) (**D**), fiber type I FCSA (**E**), fiber type IIa FCSA (**F**) and fiber type IIx FCSA (**G**) are shown. Type I fibers are stained dark (white star); type IIx fibers are stained intermediate (white arrow head) and type IIa fibers are stained light-coloured (white cross). Values are given as mean + SEM. N = 8–9 animal per group. Bar = 50 μm.

Using adenosine triphosphate (ATP) staining, we found in gastrocnemius muscle (only consisting of type IIa fibers) a pronounced 22.9% (*P* = 0.174) increase in fiber density and, however, a significant (*P* = 0.050) decrease of 21.6% in FCSA only in the male ICS group compared with control mice (Fig 1A–1D). These differences were not observed between the two female groups (Fig 1C and 1D). The female ICS group showed a significant 15.5% (*P* = 0.047) increased FCSA in comparison with the male ICS mice (Fig 1D). Moreover, like in gastrocnemius muscle, soleus muscle of male ICS mice revealed a marginal increase in fiber density and a 33.2% (*P* = 0.094) decrease–however, not significant—in FCSA in comparison with male control mice (Fig 2A–2D). Like in gastrocnemius muscle, these differences in soleus muscle were not observed between the two female groups (Fig 2C and 2D). The FCSA in soleus muscle of ICS male and female control/ICS mice was similar (Fig 2D). Fiber type I FCSA was marginally decreased in soleus of male ICS mice (Fig 2E), whereas—in parallel with the decreased FCSA–a 19.3% (*P* = 0.158) decrease of fiber type IIa (Fig 2F) and a 22.4% (*P* = 0.140) decrease of fiber type IIx (Fig 2G) in male ICS mice was observed in comparison with male control mice. These differences were not seen between the two female groups (Fig 2E–2G). The fiber type I, IIa and IIx FCSA in soleus muscle of ICS male and female control/ICS mice was similar (Fig 2E–2G).

### Differences in inflammatory (MIF^+^, IL-1β) cells in gastrocnemius and soleus muscles of male control- vs. ICS mice

**Fig 3:**
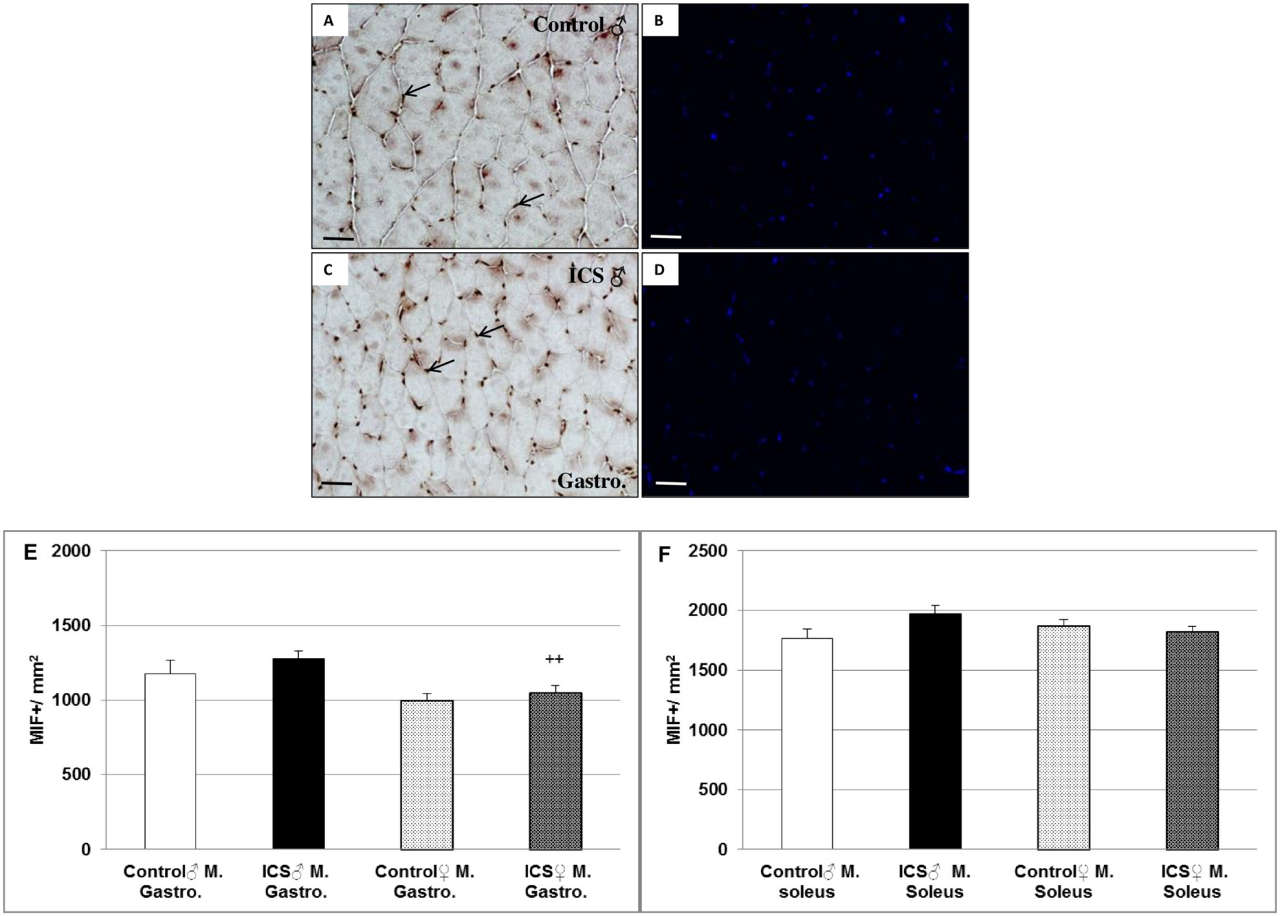
Density (n/mm^2^) of MIF^+^ cells of gastrocnemius and soleus muscles. Representative images of cross sections of male (♂) gastrocnemius muscle immunostained for MIF (**A, C**) are shown. Furthermore quantification of MIF^+^ cells of gastrocnemius (E) and soleus (F) muscles are shown. Male control (**A, B**), male ICS (**C, D**); Hoechst 33342 was used as a nuclear counterstain (**B, D**). MIF^+^ immunoreaction is indicated by black arrows (A, C) Values are given as mean + SEM; ^++^*P* <0.01 significance vs male ICS mice. N = 7–9 animals per group. Bar = 50 μm.

We hypothesized that infiltration of inflammatory cells might be responsible for the decrease in FCSA. Thus, we investigated macrophage migration inhibitory factor (MIF) as an indicator of inflammatory processes. We immunohistomorphometrically found an 8.6% (*P* = 0,296) and 11.7% (*P* = 0.067) increase of density of MIF^+^ cells in gastrocnemius (Fig 3A–3E) and soleus (Fig 3F) muscles of male ICS in comparison with male control mice (Fig 3A–3F). In contrast, the density of MIF^+^ cells in gastrocnemius and soleus muscles of female ICS mice was similar to that of female control mice (Fig 3E and 3F). However, the density of MIF^+^ cells in gastrocnemius muscle of female ICS mice was 18.1% (*P* = 0.004) lower than in gastrocnemius muscle of male ICS mice (Fig 3E). These differences seen at the protein level were not accompanied at the mRNA level (Table C in S1 File). Moreover, RNA expression measurement of another inflammatory cytokine interleukin-1 beta (IL-1β) revealed an inhibition in gastrocnemius and soleus muscles of male ICS mice, whereas a 1.5- or 3.9-fold increase was found in gastrocnemius or soleus muscles in female ICS mice (Table C in S1 File). However, the density (n/mm^2^) of IL-1β^+^ cells in gastrocnemius and soleus muscles between male/female ICS and male/female control mice was similar (Figure A in S1 File).

Interestingly, the density (n/mm^2^) of CD68^+^ macrophages in cross sections of mouse gastrocnemius and soleus muscles was similar in male/female ICS and control mice (Figure B in S1 File).

### Differences in muscle-specific E3 ubiquitin ligases (MuRF1^+^ and Fbxo32^+^) in gastrocnemius and/or soleus muscles of male control- vs. ICS mice

**Fig 4:**
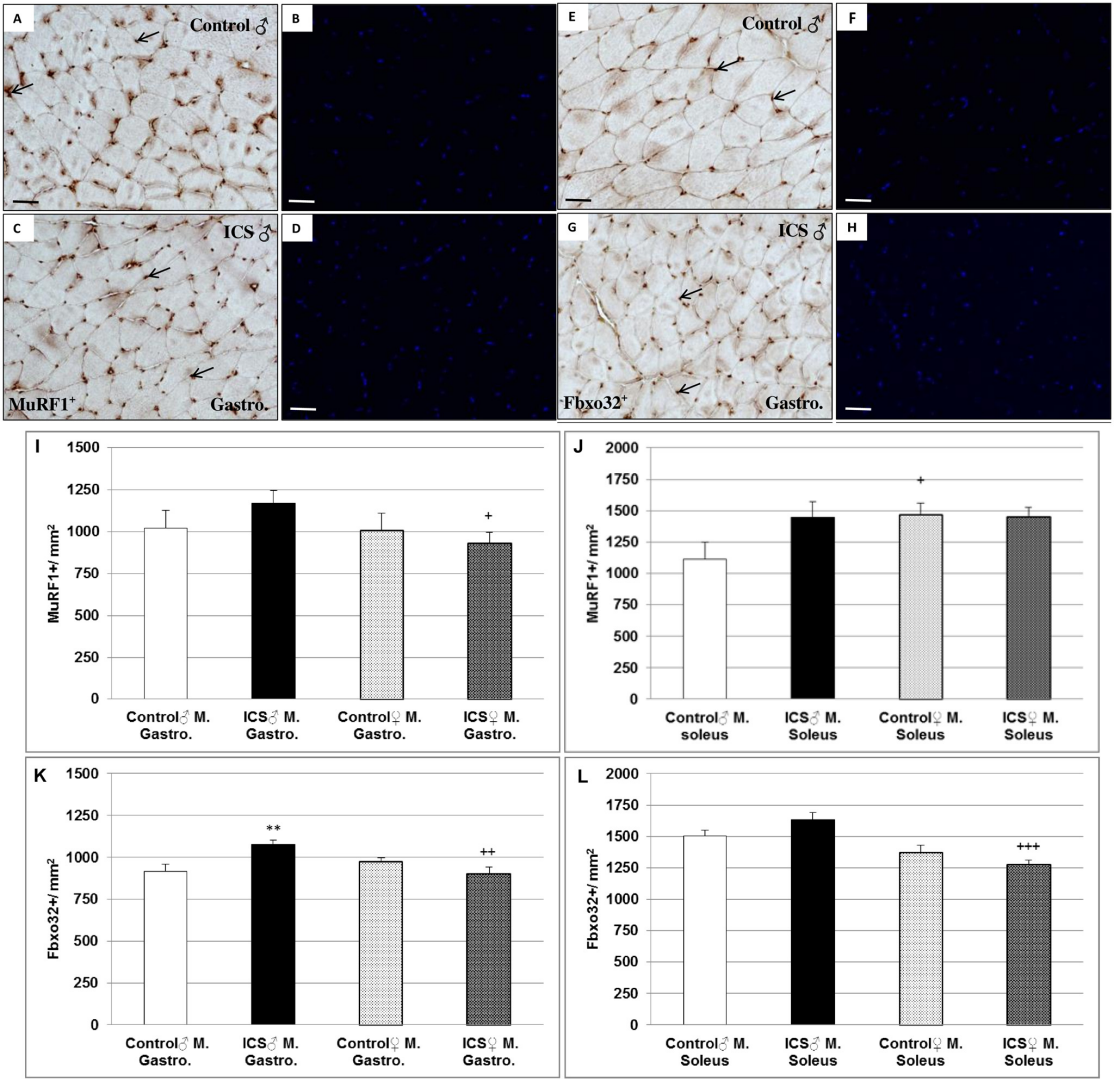
Density (n/mm^2^) of MuRF1^+^ and Fbxo32^+^ cells in mouse gastrocnemius and soleus muscles. Representative images of cross sections of male (♂) gastrocnemius muscle immunostained for MuRF1 (**A-D**) and Fbxo32 (**E-H**) are shown. Furthermore, quantification of MuRF1^+^ and Fbxo32^+^ cells in gastrocnemius (**I, K**) and soleus (**J, L**), muscles are shown. MuRF1^+^ male control (**A, B**) and male ICS (**C, D**); Fbxo32^+^ male control (**E, F**) and male ICS (**G, H**). Hoechst 33342 was used as a nuclear counterstain (**B, D, F, H**). MuRF1^+^ and Fbxo32^+^ cells are indicated by black arrows. Values are given as mean + SEM; **P < 0.01, significance vs control and ^+^*P* ≤ 0.05, ^++^*P* < 0.01 and ^+++^*P* <0.001 vs male mice. N = 7–9 animals per group. Bar = 50 μm.

Next, we investigated Muscle RING finger 1 (MuRF1 / Trim63) and muscle atrophy F-box (Fbxo32 / MAFbx / atrogin-1) known as two muscle-specific E3 ubiquitin ligases which play an important role in the atrophy of skeletal and cardiac muscle. In this context, in gastrocnemius muscle of male ICS mice we found a 14.7% (*P =* 0.087) and a 17.8% (*P* = 0.007) increase in density of MuRF1^+^ and Fbxo32^+^ cells in comparison with male control mice (Fig 4A–4I and 4K). In parallel, we found in soleus muscle of male ICS mice a 29.7% (*P* = 0.095) and a 8.9% (*P* = 0.123) increase in density of MuRF1^+^ and Fbxo32^+^ cells in comparison with male control mice (Fig 4J and 4L). However, gastrocnemius and soleus muscles of female ICS mice did not show these effects. Gastrocnemius muscle of female ICS mice showed a significant 20.8% (*P* = 0.03) and 16.2% (*P* = 0.003) decreased density of MuRF1^+^ and Fbxo32^+^ cells in comparison with male ICS mice (Fig 4I and 4K), as well as a 22.1% (*P* = 0.001) decreased density of Fbxo32^+^ cells in soleus muscle in comparison with male ICS mice (Fig 4L). In contrast, MuRF1 expression (RNA level) was decreased in gastrocnemius and soleus muscle of male ICS mice, whereas MuRF1 expression (RNA level) was about 6- and 2.3-fold increased in these muscles of female ICS mice (Table C in S1 File). In parallel, Fbxo32 expression (RNA level) was decreased in gastrocnemius and soleus muscle of male ICS mice, whereas Fbxo32 expression (RNA level) was about 4.2- and 1.5-fold increased in these muscles of female ICS mice (Table C in S1 File).

### Absence of apparent innervation defects in gastrocnemius and/or soleus muscles of control- vs. ICS mice

Acetylcholine receptors are found on the surface of muscle cells in the motor end plates. Using α-Bungarotoxin (α-BT), that binds with high affinity to the α-subunit of the nicotinic acetylcholine receptors, we found that ICS treatment had no effect on the number of motor end plates (MEP) per fiber (innervation) in gastrocnemius or soleus muscles neither in male nor in female mice (Figure C in S1 File). However, the expression (RNA level) of the cholinergic receptor, nicotinic α polypeptide 1 (Chrna) was inhibited in male/female gastrocnemius/soleus muscles after ICS treatment (Table C in S1 File).

### Differences in capillarization of gastrocnemius and/or soleus muscles of male control- vs. ICS mice

**Fig 5:**
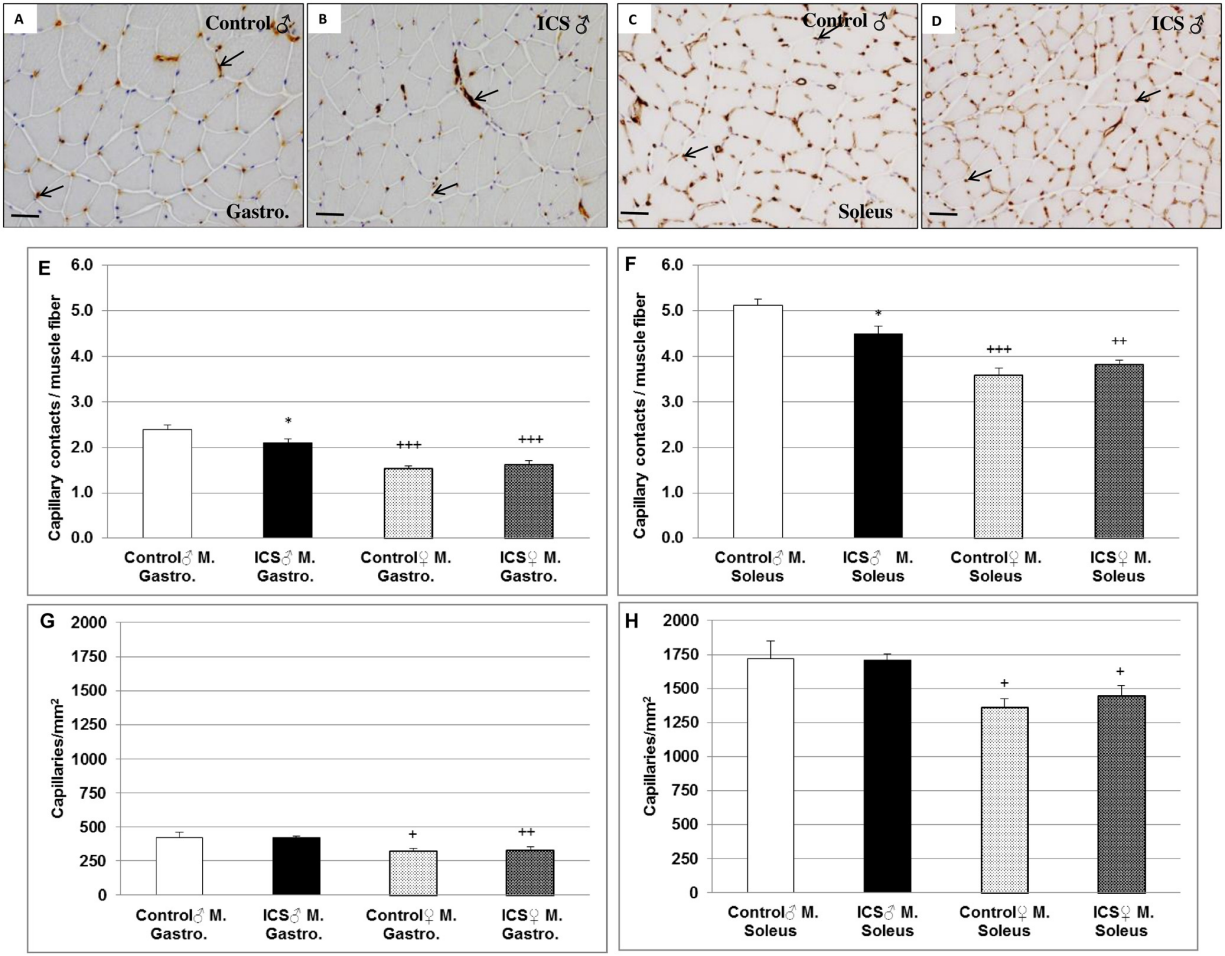
Capillary contacts/muscle fiber and density of capillaries (n/mm^2^) in cross sections of gastrocnemius and soleus muscles are shown. Representative images of cross section of male gastrocnemius (**A, B**) and soleus (**C, D**) muscles stained with the endothelial marker BSI-B4-Lectin-HRP conjugated are shown. Furthermore, quantification of capillary contacts/muscle fiber in gastrocnemius (**E**) and soleus (**F**) muscles, as well as capillary density in gastrocnemius (**G**) and soleus (**H**) muscles are shown. Male control (**A, C**) and male ICS (**B, D**), capillaries are indicated by black arrows. Values are given as mean + SEM; *P ≤ 0.05 significance vs control and ^+^*P* ≤ 0.05, ^++^ *P* < 0.01, ^+++^*P* < 0.001 vs male mice. N = 7–8 animals per group. Bar = 50 μm.

The main function of the capillaries is to distribute oxygen, nutritive materials, and hormones to muscle fibers and to collect the end products of the metabolism for further transportation to the excretory organs. Therefore, we investigated the effect of ICS on capillary contacts per fiber, as well as the density of capillaries in gastrocnemius and soleus muscles of male and female mice using the endothelial marker BSI-B4-Lectin. The capillary contacts per fiber were 12.1% (*P* = 0.044) and 12.3% (*P* = 0.010) decreased in gastrocnemius and soleus muscles of male ICS mice in comparison with the control (Fig 5A–5F). Female mice showed a less number of capillary contacts per fiber than males of the corresponding group (Fig 5E and 5F). The number of capillary contacts per fiber in soleus muscle is as twice as high as in gastrocnemius muscle (Fig 5A–5F). However, ICS had no effect on the density of capillaries in gastrocnemius and soleus muscles neither in male nor in female mice (Fig 5G and 5H); however, the female groups showed a 23.7% (control, *P* = 0.039) and 21.3% (ICS, *P* = 0.004) lower capillary density in gastrocnemius than the corresponding male groups (Fig 5G); additionally capillary density in soleus muscle of the female groups was 20.8% (control, *P* = 0.037) and 15.3% (ICS, *P* = 0.018) lower than the corresponding male groups. Moreover, the capillary density in soleus muscle of female ICS/control mice is as twice as high as in the gastrocnemius muscle of the corresponding male mice (Fig 5G and 5H).

### Correlations among density of inflammatory cells, density of cells expressing atrophy-relevant proteins, fiber density or FCSA in gastrocnemius and/or soleus muscles of male control- and ICS mice

Of note, in gastrocnemius muscle of male mice the density of inflammatory cells (IL-1β, MIF) significantly positive correlated with the density of cells expressing atrophy-relevant (MuRF1, Fbxo32) proteins, as well with the fiber density (Table 1), however significantly inverse correlated with FCSA (Table 1).

**Table 1:** Correlations of density of inflammatory (MIF^+^), atrogenic (Murf1^+^, FBXO32^+^) cells, fiber density or fiber cross sectional area in gastrocnemius and soleus muscles of male mice.

**Gastrocnemius muscle**						
		MIF^+^ (n/mm^2^)	Murf1^+^ (n/mm^2^)	Fbxo32^+^ (n/mm^2^)	Fiber Density (n/mm^2^)	Fiber cross sectional area (μm^2^)
IL-1β^+^ (n/mm^2^)	*r*	0.400	0.573	0.589	0.546	-0.179
	*P<*	0.156	0.0323	0.0266	0.0433	0.541
MIF^+^ (n/mm^2^)	*r*		0.842	0.523	0.713	-0.566
	*P<*		0.00016	0.0548	0.00419	0.0348
MuRF1^+^ (n/mm^2^)	*r*			0.745	0.744	-0.574
	*P<*			0.00221	0.00229	0.032
Fbxo32^+^ (n/mm^2^)	*r*				0.855	-0.593
	*P<*				0.0000959	0.0253
Fiber Density (n/mm^2^)	*r*					-0.767
	*P<*					0.00138
**Soleus muscle**						
IL-1β^+^ (n/mm^2^)	*r*	0.609	0.453	0.643	0.301	-0.254
	*P<*	0.0273	0.120	0.0178	0.318	0.402
MIF^+^ (n/mm^2^)	*r*		0.629	0.554	0.784	-0.522
	*P<*		0.0213	0.0495	0.00152	0.0673
MuRF1^+^ (n/mm^2^)	*r*			0.517	0.723	-0.379
	*P<*			0.0707	0.00526	0.201
Fbxo32^+^ (n/mm^2^)	*r*				0.698	-0.395
	*P<*				0.00803	0.181
Fiber Density (n/mm^2^)	*r*					-0.560
	*P<*					0.0463

FBXO32, muscle atrophy F-box (atrogin-1); IL-1ß, interleukin-1beta; MIF, macrophage migration inhibitory factor; MuRF1, Muscle RING finger 1.

Additionally, the density of cells, expressing atrophy relevant proteins also significantly positive correlated with the fiber density (Table 1) and significantly inverse correlated with FCSA (Table 1). These findings were also observed in soleus muscle of male mice, with the exception that density of inflammatory cells or cells expressing atrophy-relevant proteins insignificantly inverse correlated with FCSA (Table 1).

### Transmission electron microscopy (TEM): Increase in percentage of “damaged” mitochondria in gastrocnemius and soleus muscles of ICS- vs. control mice

**Fig 6:**
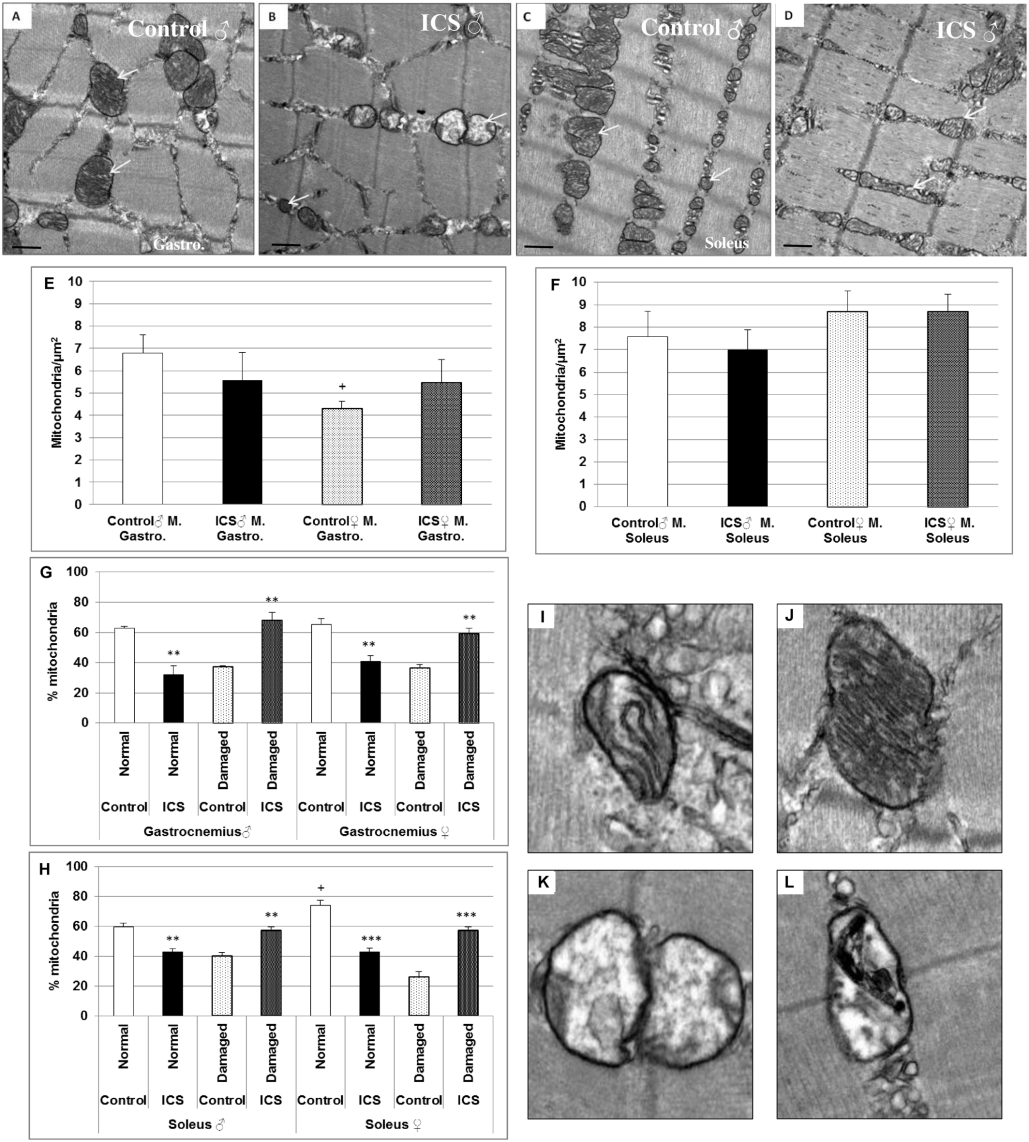
Mitochondrial density (n/mm^2^) and quality (in %) of mouse gastrocnemius and soleus muscles are shown. Representative transmission electron microscopic images of male control gastrocnemius (**A**) and soleus (**C**), male ICS gastrocnemius (**B**) and soleus ICS (**D**) muscles. Quantification of mitochondrial density and quality in gastrocnemius (**E, G**) and soleus (**F, H**) muscles are shown. Mitochondria are indicated by white arrows. Representative example of intact (**I, J**) and damaged mitochondria (**K, L**). Mitochondrium with characteristic advanced feature of autophagy indicated with a black arrow (**L**). Values are given as mean + SEM; **P < 0.01, ***P < 0.001 significance vs control and ^+^*P* ≤ 0.05, vs male mice. N = 5 animals per group. bar = 500 nm (A-D), (I-L) bar = 100 nm.

Using TEM, we analyzed the possible ICS effects on mitochondrial morphological alterations. Representative TEM images after ICS treatment showed in gastrocnemius and soleus muscles of male mice a loss of the membrane quality of the cristae and the double membrane with signaling of autophagy and increase in size (Fig 6A–6D). In gastrocnemius and soleus muscles of male ICS mice the density of mitochondria was about 17.9% (*P* = 0.494) and 8.2% (*P* = 0.713) insignificantly/tendentially decreased in comparison with control male mice (Fig 6E and 6F). However, these findings were not observed in gastrocnemius and soleus muscles of female mice (Fig 6E and 6F). Interestingly, analyses of mitochondrial quality revealed that ICS significantly affected the morphology in gastrocnemius and soleus muscles of male, but also of female mice (Fig 6G and 6H). In gastrocnemius muscles we found a significant (*P* = 0.008) 30.7% increase of “damaged” mitochondria in male ICS and a significant (*P* = 0.002) 22.5% increase in female ICS in comparison with the control (Fig 6G). Moreover, in soleus muscles we found a significant (*P* = 0.002) 16.9% increase of “damaged” mitochondria in male ICS and a significant (*P* < 0.001) 31.1% increase in female ICS in comparison with the control (Fig 6H). Representative images of severe internal mitochondrial injury in ICS mice are shown in Fig 6K and 6L. Typical characteristics of advanced mitochondrial autophagy are shown in Fig 6L. In the most severely damaged mitochondria, there is almost complete dissolution of the internal architecture (Fig 6K).

The soleus muscle of the control female group revealed a significantly (*P* = 0.023) 14.3% increased percentage of normal mitochondria, when compared with the male group (Fig 6H).

## Discussion

The aim of our study was to investigate gender, morphological and/or molecular changes in skeletal muscle to potentially deliver the basis for new strategies to pharmacologically/therapeutically treat FM by using the extensively validated ICS-induced FM mouse model.

FM has been suggested as a muscle disorder [25]. Thus, we and others [14,20] used the ICS mouse model of FM, because ICS is a useful animal model to investigate underlying mechanisms involved in the development of FM, but also to potentially evaluate possible future therapies [22] or to define gender and skeletal muscle that should be preferentially examined in future investigations.

Using ATP-staining, we found in gastrocnemius muscle (only consisting of type IIa fibers) a pronounced and in soleus muscle a marginal increase in fiber density, however, a markedly decreased FCSA exclusively in the male ICS group compared with control mice. In parallel with the decreased FCSA in soleus muscle, we could show a decrease of fiber type IIa and IIx fibers in male ICS in comparison with male control mice. These data of the FM mouse model extend clinical data most recently showing that FM patients exhibited a greater variability in fiber size and altered fiber size distribution than healthy controls [26]. Though, since clinical data derived from vastus lateralis muscle of females and findings concern differences in FCSA of gastrocnemius and soleus of male ICS mice it is tempting to speculate that vastus lateralis muscle and gastrocnemius/soleus muscles are differently affected or the response in female ICS mice is delayed in comparison with male mice. Nevertheless, the pronounced loss of FCSA in predominantly type II fibers in the ICS FM model is congruent with data of FM patients, showing an atrophy of type II fibers being described in several studies and in different muscular regions [27–29]. Moreover, altered muscle fiber size distribution has been suggested to contribute to postexertional fatigue in FM patients [26].

The major difficulty to compare the “induced” FM in an animal model with the human FM is to exactly determine the time-point when the pro-FM trigger initiated the FM syndromes, the duration and frequency of the trigger in FM patients. Moreover, the exact stimulus in humans is unknown or several stimuli do exist, probably at the same time, too. Additionally, some FM patients may have symptoms immediately after injury, whereas others reveal FM symptoms after chronic burden like stress (e. g. injury, birth, surgery, rheumatic disease, breakdown of a relationship, etc). Vice versa, in some cases, persons do not develop FM, even after any obvious trigger. In contrast, in an animal model for FM, like the ICS animal model, the trigger and the extension of the stimulus is known and investigations can be performed under controlled conditions.

Most recently it has been shown that ICS is a useful animal model to assess hypothesis about mechanisms related to the development of FM and to evaluate possible future therapies [22]. Furthermore, inflammatory processes, mediated by tissue infiltration of inflammatory cells might be responsible for the decreased FCSA. In this context, a marginal increase of density of MIF^+^ cells in gastrocnemius and soleus muscles of male ICS in comparison with male control mice was found. According to our data and to the most recent findings that in FM patients no signs of inflammation were observed in muscle [26], inflammation seems to play a minor role for the symptoms of FM in ICS mice also. Thus, other factors should be taken into account. For atrophy of skeletal and cardiac muscle the two muscle-specific E3 ubiquitin ligases MuRF1 and Fbxo32 play an important role. These two “atrogenes” are expressed at relatively low levels in resting skeletal muscle, however, their expression rapidly increases after the onset of several stressors, prior to muscle loss [30]. We here show an increase in density of MuRF1^+^ and Fbxo32^+^ cells in gastrocnemius and soleus muscles of male ICS mice in comparison with male control mice, indicating that induction of these two “atrogenes” might be responsible for the reduction of FCSA in skeletal muscles of male ICS mice. Together with data of a most recent study conducted in a FM rat model [31], these findings provide direct evidence that exposure to cold increases proteolytic processes in skeletal muscles *via* MuRF1 and Fbxo32.

Because we show that FCSA of gastrocnemius and soleus muscles of male ICS mice was markedly decreased and it has been suggested that the problems of FM patients might be due to a consequence of the modified pattern of motor unit innervation [32], we investigated the innervation of the skeletal muscles using α-Bungarotoxin. Yet, we found that ICS had no visible or numerical effect on the number of motor end plates per fiber (innervation) in gastrocnemius or soleus muscles neither in male nor in female mice. These data indicate that besides inflammatory effects also motor unit innervation seem to play a minor role in ICS mice and FM patients as well.

To investigate oxygen delivery to the muscle in ICS and control mice, the number of capillaries per fiber and the capillary density (capillaries per area tissue) were determined. We here show that the capillary contacts per fiber were significantly decreased in gastrocnemius and soleus muscles of male ICS in comparison with control mice. Moreover, the number of capillary contacts per fiber in soleus muscle is as twice as high as in gastrocnemius muscle. However, ICS had no effect on the density of capillaries in gastrocnemius and soleus muscles. These data indicate that ICS reduces the distribution of oxygen, nutritive materials, and hormones to muscle fibers as well as decreases the collection and transport of end products of the metabolism to excretory organs. However, the protection of soleus muscle against these impairments seem to be as twice as high in comparison with gastrocnemius muscle. Especially in skeletal muscle consisting of type II fibers, a deterioration of the microcirculation may result in lower oxygen delivery and waste product clearance, which may directly contribute to pathologic distribution of tissue oxygen as well as to pain and fatigue in FM [26,33]. Therefore, the decreased number of capillary contacts to fiber in addition with the decreased FCSA (this study) may account for post-exertional fatigue in FM as described by others, showing that number of capillaries per fiber was significantly lower among patients with FM compared to healthy controls [26]. Our data may also explain abnormalities in skeletal muscles as changes of intramuscular microcirculation or muscle energy metabolism that have been demonstrated in FM patients [33].

Here we show, for the first time, that in gastrocnemius muscle of male mice the density of inflammatory cells (IL-1β, MIF) positively correlated with the density of cells expressing atrophy-relevant (MuRF1, Fbxo32) proteins, as well as with the fiber density; yet inversely correlated with FCSA. Additionally, the density of cells, expressing atrophy relevant proteins also positively correlated with the fiber density and inversely correlated with FCSA. These findings are congruent with correlations of these parameters in soleus muscle, with the exception that density of inflammatory cells or cells expressing atrophy-relevant proteins did not inversely correlate with FCSA. Our data indicate that in skeletal muscles of male ICS mice inflammation and/or atrophy-relevant proteins seem to be associated with the regulation of fiber density and / or FCSA. Thus, we suggest to analyse these possible correlations also in muscle biopsies of FM patients. Moreover, the missing of significant correlations between inflammation and / or atrophy-relevant proteins and FCSA in soleus muscle may indicate a delayed regulation in this predominantly of type I fibers consisting muscle and in skeletal muscles of female ICS mice as well.

Moreover, in our study TEM revealed a loss of the membrane quality of the cristae and the double membrane with signaling of autophagy and increase in size. Furthermore, TEM showed that in gastrocnemius and soleus muscles of male ICS mice the density of mitochondria was decreased in comparison with control male mice; additionally, analyses of mitochondrial quality revealed that ICS affected the morphology in gastrocnemius and soleus muscles of male and female mice. These data are congruent with findings of FM patients showing that number, size, shape, function and organization of mitochondria in the muscle tissue is substantially altered in comparison with controls [25,26,34,35]. These morphological and numerical mitochondrial abnormalities, with potential impact on function, may not only impair oxidative capacity but also atrophy of individual fibers.

Finally, understanding of these above described defects in skeletal muscles of ICS mice as well as in muscle of FM patients provides valuable insight with regard to future treatment [26].

## Conclusion

The ICS-induced decrease of FCSA mainly concerns gastrocnemius muscle of male mice due to an increase of inflammatory and atrogenic cells. In soleus muscle of male ICS and soleus/gastrocnemius muscles of female ICS mice morphological alterations seem to occur not at all or delayed. ICS induces long-lasting muscle hyperalgesia and allodynia; thus, we suggest to investigate morphological and/or molecular alterations at different time-points (up to two weeks) after ICS.

## Supporting Information

S1 File **Table A.** Primary antibodies used in the study. **Materials and methods A.** α-Bungarotoxin (α-BT) histochemistry. **Table B.** Primers used for real time qRT-PCR. **Table C.** Effect of ICS on mRNA expression of relevant genes analyzed by qRT-PCR. **Fig A.** Density (n/mm^2^) of IL-1β+ cells in gastrocnemius (A) and soleus (B) muscles. Values are given as mean + SEM; ++P<0.01, significance vs male mice. N = 6–9 animals per group. **Fig B.** Density (n/mm^2^) of CD68+ macrophages in cross sections of mouse gastrocnemius and soleus muscles are shown. Representative images of cross section of gastrocnemius muscle immunostained for CD68 (A-B) and quantification of CD68+ macrophages in gastrocnemius (C) and soleus (D) muscles are shown. CD68 immunoreactive macrophages are indicated by black arrows. Values are given as mean + SEM; +P<0.05 significance vs male. N = 7–9 animals per group. Bar = 50 μm. **Fig C.** Density of motor end plates (MEP) (MEP/fiber) in cross sections of mouse gastrocnemius (A) and soleus (B) muscles are shown. Values are given as mean + SEM. N = 5 animals per group.
(DOCX)

## Abbreviations

ACR: American college of rheumatology
ATP: Adenosine triphosphate
DAB: 3’ diaminobenzidine solution
Fbxo32: F-Box Protein 32
FCSA: fibre cross selectional area
FM: fibromyalgia
HRP: horseradish peroxidase
ICS: intermittent cold stress
IHC: immunohistochemistry
MEP: motor end plates
MIF: macrophage migration inhibitory factor
MuRF1: muscle ring finger 1
PFA/PBS: paraformaldehyde/phosphate buffered solution
ROS: reactive oxygen species
RT: room temperature
SART: specific alteration rhythm of temperature
SEM: standard error of the mean
TEM: transmission electron microscopy
UPS: ubiquitin proteasome system
