# Supplementary material for: Morphological Alterations in Gastrocnemius and Soleus Muscles in Male and Female Mice in a Fibromyalgia Model
Source: PLoS One. 2016 Mar 17;11(3):e0151116. doi: 10.1371/journal.pone.0151116 (PMC4795636; doi:10.1371/journal.pone.0151116)
Supplement: S1 File — Table A. Primary antibodies used in the study. Materials and methods A. α-Bungarotoxin (α-BT) histochemistry. Table B. Primers used for real time qRT-PCR. Table C. Effect of ICS on mRNA expression of relevant genes analyzed by qRT-PCR. Fig A. Density (n/mm2) of IL-1β+ cells in gastrocnemius (A) and soleus (B) muscles. Values are given as mean + SEM; ++P<0.01, significance vs male mice. N = 6–9 animals per group. Fig B. Density (n/mm2) of CD68+ macrophages in cross sections of mouse gastrocnemius and soleus muscles are shown. Representative images of cross section of gastrocnemius muscle immunostained for CD68 (A-B) and quantification of CD68+ macrophages in gastrocnemius (C) and soleus (D) muscles are shown. CD68 immunoreactive macrophages are indicated by black arrows. Values are given as mean + SEM; +P<0.05 significance vs male. N = 7–9 animals per group. Bar = 50 μm. Fig C. Density of motor end plates (MEP) (MEP/fiber) in cross sections of mouse gastrocnemius (A) and soleus (B) muscles are shown. Values are given as mean + SEM. N = 5 animals per group. (DOCX) [file pone.0151116.s001.docx]

**Supporting Information**

**Table A.** Primary antibodies used in the study

| **Antigen** | **Antibody name and dilution** | **Cat. no.** | **company** |
| --- | --- | --- | --- |
| CD68 | Monoclonal rat anti- mouse CD68. 1:50 | MCA1957 | AbD Serotec, Kidlington, UK |
| Fbxo32 | Polyclonal rabbit anti- Fbx32. 1:200 | ab74023 | Abcam plc., Cambridge, UK |
| IL1- β | Polyclonal rabbit anti- Interleukin 1- β. 1:100 | ab9722 | Abcam plc., Cambridge, UK |
| MIF | Polyclonal rabbit anti- Macrophage migration inhibitory factor. 1:100 | ab7207 | Abcam plc., Cambridge, UK |
| Trim63  (MuRF1) | Polyclonal rabbit anti- MuRF1 (H- 145). 1:100 | sc-32920 | Santa Cruz Biotechnology Inc.; Dallas, USA |

**Materials and methods A.**

**α-Bungarotoxin (α-BT) histochemistry:**

To analyze neuromuscular junctions, cryo cross-sections (6-7 µm) of gastrocnemius and soleus muscles were fixed with 4% PFA/PBS 10 min, endogenous peroxidase was blocked with 3% H_2_O_2_ and afterwards incubated overnight with 2 ng/µl α-Bungarotoxin, Biotin-XX-conjugated (Life Technologies GmbH, Darmstadt, Germany). After washing of the sections with PBS, unspecific binding sites were blocked with 2% BSA. Thereafter sections were incubated with Streptavidin-HRP-conjugated (Jackson ImmunoResearch Laboratories. Inc., West Grove, USA) and with DAB solution (Roche Diagnostics). Nuclei were counterstained with Mayer's Hematoxylin (Carl Roth GmbH. Neuromuscular junctions were identified in cross-sections by brown staining and quantified using the software AxioVision Release 4.8.2 (Carl Zeiss GmbH). The average neuromuscular junctions per area (number of α-bungarotoxin^+^/mm^2^) was calculated for each muscle cross-section.

**Table B.** Primers used for real time qRT-PCR

| **Primer** | **Symbol** | **Amplicon Length (bp)** | **Cat. no.** |
| --- | --- | --- | --- |
| Actin, beta | Actb | 77 | QT01136772 |
| Glyceraldehyde-3-phosphate dehydrogenase | Gapdh | 144 | QT01658692 |
| Interleukin 1- β | Il1β | 150 | QT01048355 |
| Cholinergic receptor, nicotinic,α polypeptide 1 (muscle) | Chrna1 | 94 | QT00109942 |
| F-box protein 32 | Fbxo32 | 103 | QT00158543 |
| TATA box binding protein | Tbp | 114 | QT00198443 |
| Tripartite motif-containing 63 (Murf-1) | Trim63 | 116 | QT00291991 |

**Table C.** Effect of ICS on mRNA expression of relevant genes analyzed by qRT-PCR

|  | **ICS♂ Gastro.** | **ICS♀ Gastro.** | **ICS♂ Soleus** | **ICS♀ Soleus** |
| --- | --- | --- | --- | --- |
| **MIF** | 0.90 | 0.66 | 0.76 | 1.23 |
| **IL-1ß** | 0.65 | 1.53 | 0.85 | 3.88 |
| **MuRF1** | 0.67 | 6.04 | 0.45 | 2.29 |
| **Fbxo32** | 0.88 | 4.15 | 0.83 | 1.53 |
| **Chrna** | 0.37 | 0.49 | 0.50 | 0.79 |

**ICS, intermittent cold stress; Gastro., gastrocnemius muscle; soleus, soleus muscle; ♂, male; ♀, female (control=1).**


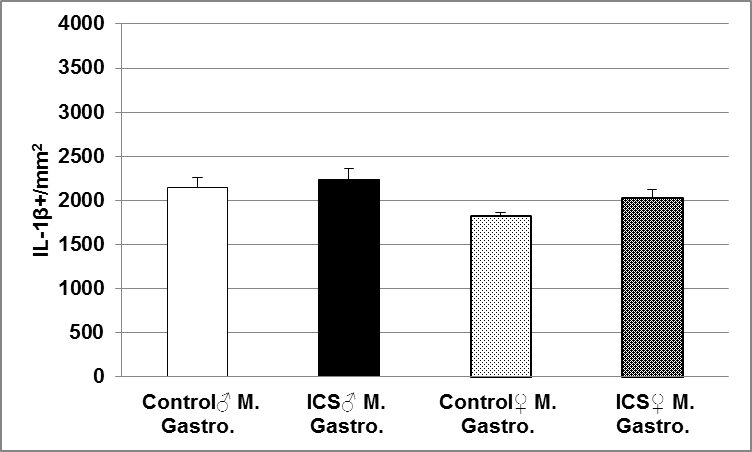

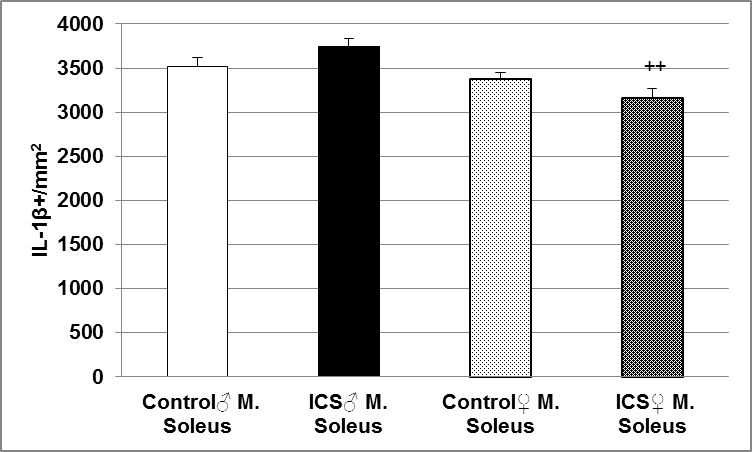


**A**

**B**

**Figure A**. Density (n/mm²) of IL-1β^+^ cells in gastrocnemius (A) and soleus (B) muscles. Values are given as mean + SEM; ^++^P<0.01, significance vs male mice. N= 6-9 animals per group.

**Control ♂**

**ICS ♂**

**Gastro.**

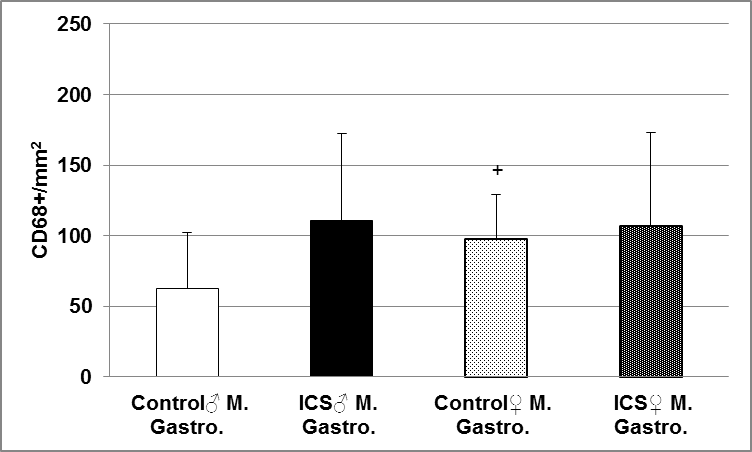

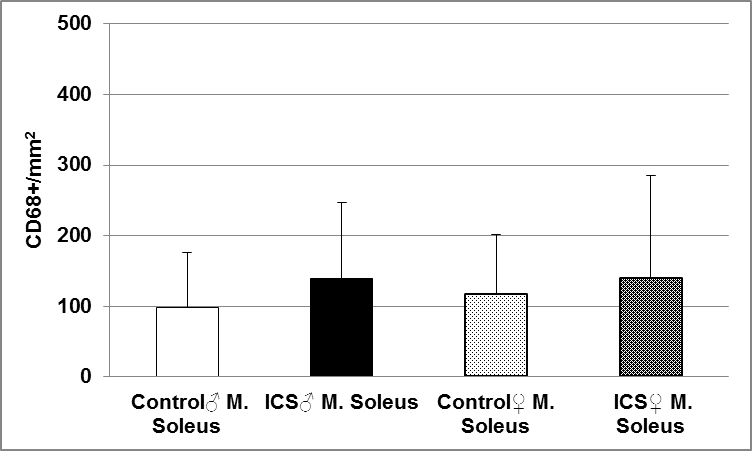


**C**

**D**

**Figure B**. Density (n/mm²) of CD68^+^ macrophages in cross sections of mouse gastrocnemius and soleus muscles are shown. Representative images of cross section of gastrocnemius muscle immunostained for CD68 (A-B) and quantification of CD68^+^ macrophages in gastrocnemius (C) and soleus (D) muscles are shown. CD68 immunoreactive macrophages are indicated by black arrows. Values are given as mean + SEM; ^+^P<0.05 significance vs male. N= 7-9 animals per group. Bar = 50 µm.


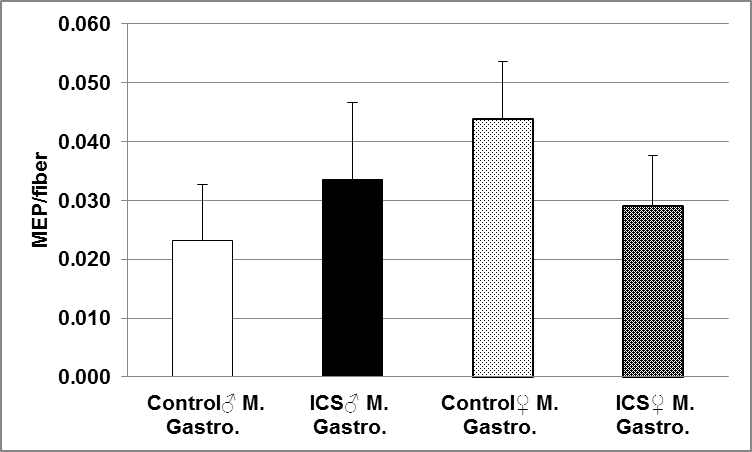

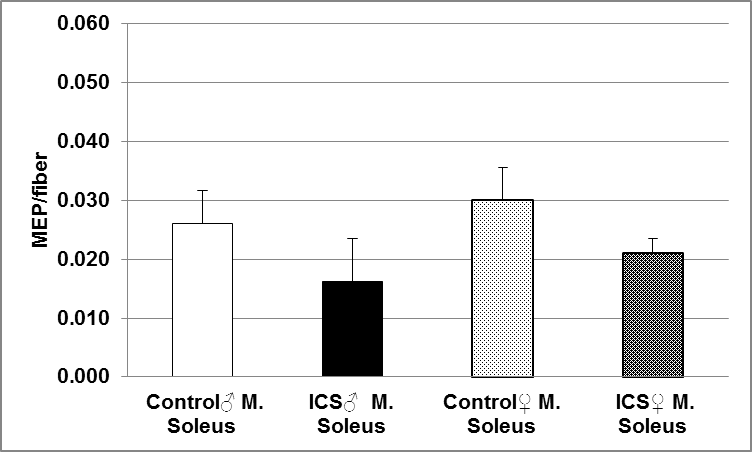


**B**

**A**

**Figure C.** Density of motor end plates (MEP) (MEP/fiber) in cross sections of mouse gastrocnemius (A) and soleus (B) muscles are shown. Values are given as mean + SEM. N= 5 animals per group.
